# A Clinical, Pathological, and Experimental Study of Fracture of the Lower End of the Radius

**Published:** 1898-12

**Authors:** 


A CLINICAL, PATHOLOGICAL, AND EXPERIMENTAL STUDY OF FRACTURE OF THE LOWER END OF THE RADIUS. With Displacement of the Carpal Fragment Forward on Anterior Surface of the Wrist. By John B. Roberts, A. M., M. D., Professor of Anatomy and Surgery in the Philadelphia Polyclinic; Professor of Surgery in the Woman's Medical College of Pennsylvania; Surgeon to the Methodist and Jewish Hospitals. With thirty-three illustrations. Published by P. Blakiston, Son & Co., Philadelphia. 1897. Seventy-six pages. Price. cloth, $1.

The above is the somewhat prolix title page of a very valuable little monograph on Colle's fracture of the radius, with forward displacement of the lower fragment. This condition according to the text-books is quite rare, and necessarily so from the fact that the fracture is almost invariably caused by a fall upon the open palm.

If, however, the injury arises from some other cause, it would seem that forward displacement would be as likely to occur as backward, as the displacement is not due to muscular action but to the impact of the injury. This theory is well established by Prof. Roberts, by citing many recorded cases, anatomical and museum specimens, skiagraphs and laboratory experiments. The monograph closes with a few pages devoted to the symptoms, diagnosis and treatment of this peculiar fracture. I. J. J.

